# A colorectal cancer prediction model using traditional and genetic risk scores in Koreans

**DOI:** 10.1186/s12863-015-0207-y

**Published:** 2015-05-09

**Authors:** Keum Ji Jung, Daeyoun Won, Christina Jeon, Soriul Kim, Tae Il Kim, Sun Ha Jee, Terri H Beaty

**Affiliations:** Department of Public Health, Graduate School, Yonsei University, Seoul, South Korea; The Catholic University of Korea, Seoul Saint Mary’s Hospital, Seoul, South Korea; Institute for Health Promotion and Department of Epidemiology and Health Promotion, Graduate School of Public Health, Yonsei University, 50 Yonse-ro, Seodaemun-gu, Seoul South Korea; Division of Gastroenterology, Department of Internal Medicine, Yonsei University College of Medicine, Seoul, South Korea; Johns Hopkins Bloomberg School of Public Health, Baltimore, MD USA

**Keywords:** Single nucleotide polymorphisms, Gene-traditional risk score, Colorectal cancer

## Abstract

**Background:**

Genome-wide association studies have identified numerous single nucleotide polymorphisms (SNPs) as associated with colorectal cancer (CRC) risk in populations of European descent. However, their utility for predicting risk to CRC in Asians remains unknown. A case-cohort study (random sub-cohort N = 1,685) from the Korean Cancer Prevention Study-II (KCPS-II) (N = 145,842) was used. Twenty-three SNPs identified in previous 47 studies were genotyped on the KCPS-II sub-cohort members. A genetic risk score (GRS) was calculated by summing the number of risk alleles over all SNPs. Prediction models with or without GRS were evaluated in terms of the area under the receiver operating characteristic curve (AUROC) and the continuous net reclassification index (NRI).

**Results:**

Seven of 23 SNPs showed significant association with CRC and rectal cancer in Koreans, but not with colon cancer alone. AUROCs (95% CI) for traditional risk score (TRS) alone and TRS plus GRS were 0.73 (0.69–0.78) and 0.74 (0.70–0.78) for CRC, and 0.71 (0.65–0.77) and 0.74 (0.68–0.79) for rectal cancer, respectively. The NRI (95% CI) for a prediction model with GRS compared to the model with TRS alone was 0.17 (-0.05-0.37) for CRC and 0.41 (0.10–0.68) for rectal cancer alone.

**Conclusion:**

Our results indicate genetic variants may be useful for predicting risk to CRC in the Koreans, especially risk for rectal cancer alone. Moreover, this study suggests effective prediction models for colon and rectal cancer should be developed separately.

**Electronic supplementary material:**

The online version of this article (doi:10.1186/s12863-015-0207-y) contains supplementary material, which is available to authorized users.

## Background

According to the Korean National Cancer Center, the incidence of colorectal cancer (CRC), the 3^rd^ most common cancer in Korea, has increased from 21.2/100,000 people in 1999 to 39.0/100,000 people in 2011 [1]. Steady increases in the incidence of CRC should be expected, partly due to environmental factors such as increased Western dietary patterns. Early discovery of high-risk groups could be helpful in managing risk factors and ultimately in reducing CRC incidence and mortality [2].

Previous studies have proposed CRC prediction models but these attained only limited predictive power [3,4]. Some models reflect only one aspect of the associated risk factors and failed to incorporate both the genetic and traditional risk factors (including environmental factors) of CRC [3-5]. Moreover, many previous models did not distinguish between the colon and rectal cancer, which are distinct by anatomic sites and other characteristics [2,6]. In fact, previous publications have reported colon and rectal cancer show different associations with traditional risk factors [7-9]. Therefore, to develop more effective prediction models, we should 1) include information on both genetic and traditional risk factors, and 2) distinguish between colon and rectal cancers.

For our CRC predictive model, the most appropriate traditional risk factors were determined from a prospective cohort study of the general Korean population. Also, after incorporating genetic factors into the model, its utility was carefully evaluated. Our study provides evidence that considering genetic factors as well as traditional risk factors in risk prediction models can improve their utility.

## Results

We attained 633,210 person-years (PY) after following 145,842 study subjects through December 2012. During the follow-up period, 258 CRC patients were verified from the National Cancer Center cancer registry database. Overall incidence rate per 100,000 PY was 40.7.

Table 1 shows the characteristics of all study participants. Participants from the KCPS-II cohort and sub-cohort had similar characteristics of age, sex, BMI, smoking status, alcohol drinking, exercise, and family history. In each cohort, the case group was older and had higher BMI and fasting blood glucose than did the control group. Also, in each cohort, the patient group showed higher rates of smoking and more cases reported a family history of CRC.

**Table 1:** **General characteristics of study participants: The Korean Cancer Prevention Study-II and the KCPS-II sub-cohort**

		**KCPS-II cohort (Whole participants)**			**KCPS-II sub-cohort (Case-cohort design)**		
		**CRC**	**No CRC**	***P***	**CRC**	**No CRC**	***P***
N		258	145,584		173	1,514	
Age, year		50.7 ± 10.5	41.1 ± 10.3	<0.001	49.7 ± 10.9	40.1 ± 9.4	<0.001
Sex, % of female		24.1	37.9	<0.001	25.0	37.6	0.001
Body mass index, kg/m^2^		24.3 ± 2.7	23.6 ± 3.2	<0.001	24.3 ± 2.7	23.5 ± 3.2	0.001
Fasting blood glucose, mg/dL		99.0 ± 25.3	91.0 ± 19.0	<0.001	98.2 ± 27.8	90.1 ± 17.9	<0.001
Total cholesterol, mg/dL		197.7 ± 37.1	189.0 ± 33.8	0.002	195.6 ± 36.9	189.3 ± 41.8	0.037
Systolic blood pressure, mmHg		123.4 ± 16.3	117.9 ± 14.4	<0.001	121.8 ± 14.7	117.6 ± 14.3	0.037
Smoking status, %	Ex	31.0	17.7	<0.001	29.6	15.8	<0.001
	Current	29.9	29.2		30.7	30.2	
Alcohol drinking (yes), %		73.6	74.0	0.881	74.4	76.7	0.496
Exercise (yes), %		63.2	59.6	0.228	62.5	61.8	0.850
Family history of CRC (yes), %		5.0	2.3	0.005	5.1	2.0	0.011

Values are mean ± standard deviation (SD) for continuous data.

Body mass index (BMI) = weight in kilograms divided by height in meters squared.

CRC: Colorectal cancer, KCPS-II: The Korean Cancer Prevention Study-II.

Table 2 shows the estimated hazards ratio (HR) of various factors contributing to the risk of CRC. Each cohort showed similar findings between participants in the whole KCPS-II cohort and the sub-cohort participants. Age, sex, fasting serum glucose, smoking status, exercise, and family history were ultimately selected as predictors for CRC.

**Table 2:** **Hazard ratios for risk factors on risk of CRC: The Korean Cancer Prevention Study-II and the KCPS-II sub-cohort**

		**KCPS-II cohort (Whole participants)**	**KCPS-II sub-cohort (Case-cohort design)**
**Traditional risk factors**		**HR (95% CI)**	**HR (95% CI)**
Age, year		1.07 (1.06-1.08)	1.08 (1.07-1.10)
Sex (female)		0.65 (0.45-0.92)	0.71 (0.50-1.11)
Log (fasting serum glucose), mg/dL		1.81 (0.99-3.30)	2.16 (0.96-4.83)
Smoking status	Ex-smoker	1.45 (1.03-2.05)	1.74 (1.12-2.72)
	Current smoker	1.28 (0.90-1.83)	1.40 (0.91-2.17)
Exercise (yes)		0.91 (0.70-1.17)	0.68 (0.49-0.94)
Family history of CRC (yes)		2.40 (1.34-4.30)	3.49 (1.70-7.17)
Per 1 SD of TRS increase		1.34 (1.29-1.39)	1.30 (1.24-1.36)

CRC: colorectal cancer, HR: hazard ratios, CI: confidence interval, SD: standard deviation, TRS: traditional risk score,

TRS combined information on above 6 risk factors: age, sex, fasting serum glucose, exercise, and family history of CRC.

KCPS-II: The Korean Cancer Prevention Study-II.

Table 3 shows allelic association with CRC, colon, and rectal cancer, respectively. Depending on the cancer location (colon or rectum), each SNP showed a different pattern of association. A total of 5 out of 23 SNPs showed significant association only with rectal cancer, but not on colon cancer. A total of 2 out of 23 SNPs showed a positive association across both colon and rectum cancer, although it was only moderately significant.

**Table 3:** **Allelic odds ratios for subtype of CRC in the Korean Cancer Prevention Study II sub-cohort**

	**Reference number in Additional file** **2** **: Table S1**				**Colorectal cancer**	**Colon cancer**	**Rectal cancer**
SNPs*		**Chr.**	**RA**	**RAF**	**OR (95% CI)**	**OR (95% CI)**	**OR (95% CI)**
rs3802842	4,7,19,29,32,34, 36	11	C	0.40	**1.46 (1.14-1.86)**	**1.30 (0.93-1.81)**	**1.50 (1.10-2.04)**
rs4444235	4,7,32,38	14	C	0.52	1.02 (0.80-1.29)	1.01 (0.73-1.40)	1.03 (0.76-1.40)
rs4939827	7,10,29,30,34,41,42,43,44,45,46	18	T	0.22	**1.32 (1.01-1.71)**	1.04 (0.71-1.52)	**1.55 (1.11-2.16)**
rs6983267	7,16,17,18,19,20,21,22,23,24,25,26,27,28	8	G	0.43	**1.14 (0.91-1.43)**	0.85 (0.61-1.17)	**1.46 (1.08-1.97)**
rs10505477	11,12,13,14,15	8	G	0.43	**1.15 (0.92-1.45)**	0.88 (0.64-1.21)	**1.44 (1.06-1.94)**
rs10795668	7,20,30,31,32,33,34	10	G	0.64	**1.20 (0.92-1.55)**	0.93 (0.66-1.32)	**1.45 (1.03-2.05)**
rs11169552	1	12	T	0.34	0.98 (0.76-1.25)	1.05 (0.75-1.47)	0.93 (0.67-1.28)
rs6687758	1,2	1	G	0.29	0.96 (0.74-1.25)	1.14 (0.80-1.63)	0.85 (0.60-1.20)
rs7014346	29	8	G	0.69	0.94 (0.73-1.21)	1.08 (0.76-1.54)	0.86 (0.62-1.18)
rs11903757	3	2	T	0.96	0.79 (0.45-1.42)	0.75 (0.35-1.63)	0.91 (0.42-1.96)
rs3217810	3	12	C	0.95	0.98 (0.19-5.21)	0.44 (0.08-2.45)	NE
rs10411210	20,28	19	T	0.18	0.91 (0.66-1.25)	0.82 (0.52-1.30)	1.00 (0.67-1.50)
rs961253	4,7,11,19,20,34, 38,47	20	A	0.10	**1.38 (0.97-1.97)**	**1.19 (0.72-1.98)**	**1.45 (0.93-2.26)**
rs6691170	1,2,4	1	T	0.09	NE		
rs9929218	20,21,31,38,40	16	A	0.15	**1.21 (0.87-1.68)**	**1.16 (0.74-1.83)**	**1.20 (0.78-1.82)**
rs10911251	3	1	C	0.46	1.01 (0.80-1.29)	0.80 (0.58-1.12)	1.22 (0.90-1.66)
rs7758229	10	6	T	0.22	1.06 (0.80-1.41)	0.91 (0.60-1.37)	1.20 (0.84-1.71)
rs59336	3	12	T	0.63	0.94 (0.73-1.20)	0.79 (0.57-1.12)	1.09 (0.79-1.52)
rs3217901	37	12	G	0.65	1.10 (0.87-1.39)	**1.51 (1.08-2.11)**	0.83 (0.61-1.12)
rs10936599	1,4,5,6,7,8	3	T	0.61	1.09 (0.87-1.38)	1.07 (0.77-1.48)	1.11 (0.81-1.50)
rs647161	9	5	C	0.69	0.95 (0.73-1.22)	0.72 (0.51-1.01)	1.25 (0.88-1.77)
rs7136702	1	12	T	0.53	1.08 (0.85-1.37)	1.10 (0.79-1.54)	1.02 (0.75-1.39)
rs4779584	4,19,20,30,32,33,36,39	15	T	0.84	0.97 (0.70-1.34)	0.91 (0.58-1.43)	1.02 (0.67-1.55)

CRC: colorectal cancer, Chr.: chromosome, RA: risky allele, RAF: risky allele frequency, OR: odds ratio, CI: confidence interval, NE: not estimated due to small number, SNP with ORs in bold were selected for genetic risk score calculations.

*List of references and detailed information were summarized in Additional file 2: Table S1.

In this study, the GRS was based on 7 SNPs (rs3802842, rs4939827, rs6983267, rs10505477, rs10795668, rs961253, and rs9929218). Overall these GRS followed a normal distribution (data not shown).

Table 4 shows the predictive power of models incorporating GRS with TRS for CRC, and rectal cancer using both the ROC area and NRI. AUROC (95% CI) for TRS alone was 0.73 (0.69-0.78) for CRC, and 0.71 (0.65–0.77) for rectal cancer alone. The AUROC (95% CI) for the combined model with both TRS and GRS was increased, especially for rectal cancer [0.74 (0.68-0.79)]. NRI (95% CI) for the model with GRS compared to the model with only TRS was 0.17 (-0.05–0.37) for CRC, and 0.41 (0.10–0.68) for rectal cancer. Table 4 also shows the risk of CRC and rectal cancer alone after dividing GRS into quartiles. Compared with participants in the lowest quartile, those with the highest quartile of GRS had a 2.65-fold higher risk for CRC and a 10.83-fold higher risk for rectal cancer alone, respectively.

**Table 4:** **Area under receiver operating characteristic curve by subtype of CRC: Korean Cancer Prevention Study II sub-cohort**

		**Colorectal cancer**		**Colon cancer**		**Rectal cancer**	
		**HR (95% CI)**	HR (95% CI)*	**HR (95% CI)**	HR (95% CI)*	**HR (95% CI)**	HR (95% CI)*
TRS	Q1	1.00	1.00	1.00	1.00	1.00	1.00
	Q2	1.97 (0.95-4.11)	2.03 (0.98-4.22)	1.60 (0.52-4.94)	1.60 (0.52-4.95)	2.28 (0.87-6.01)	2.40 (0.91-6.31)
	Q3	2.57 (1.29-5.14)	2.62 (1.31-5.24)	2.19 (0.76-6.32)	2.16 (0.75-6.24)	2.88 (1.15-7.24)	3.02 (1.20-7.59)
	Q4	11.29 (6.06-21.1)	11.54 (6.19-21.5)	13.33 (5.31-33.5)	13.27 (5.29-33.3)	10.16 (4.36-23.7)	10.59 (4.54-24.7)
GRS	Q1		1.00		1.00		1.00
	Q2		1.42 (0.77-2.61)		0.95 (0.47-1.91)		3.95 (0.93-16.84)
	Q3		1.68 (0.92-3.07)		0.77 (0.37-1.59)		7.06 (1.70-29.28)
	Q4		2.65 (1.43-4.91)		1.17 (0.55-2.50)		10.83 (2.58-45.40)
AUROC		0.73 (0.69-0.78)^†^	0.74 (0.70-0.78)	0.76 (0.70-0.83)^†^	0.75 (0.69-0.81)	0.71 (0.65-0.77)^†^	0.74 (0.68-0.79)
NRI		-	0.17 (-0.05-0.37)		−0.17 (-0.33-0.21)	-	0.41 (0.10-0.68)
P for NRI			0.108		0.688		0.008

CRC: colorectal cancer, TRS: traditional risk score, GRS: genetic risk score, HR: hazard ratio, CI: confidence interval, AUROC: Area under receiver operating characteristic curve, NRI: net reclassification index.

*Combined model, ^†^AUROC for TRS alone, AUROC for TRS + GRS.

**Figure 1:**
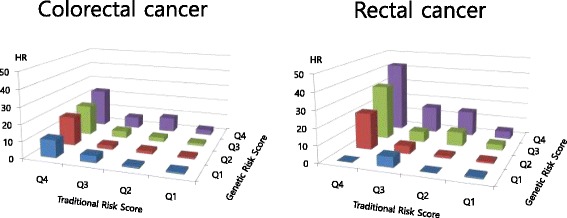
Combined effect of traditional risk score and genetic risk score on colorectal cancer: Korean Cancer Prevention Study-II.

Figure 1 shows the combined risk of CRC and rectal cancer separately after dividing each GRS and TRS into quartiles. As the GRS increased into quartile 4 (Q4), the CRC risk increased. Also, as the TRS increased in quartile 4 (Q4), the CRC risk increased even more. Participants with TRS and GRS in the highest quartile (Q4) were determined to have about 25 times higher risk of CRC than those with TRS and GRS in the lowest quartile (Q1). Likewise, participants with TRS and GRS in the highest quartile (Q4) were determined to have about 40 times as much risk of rectal cancer compared to those with TRS and GRS in the lowest quartile (Q1).

## Discussion

### Gene-based prediction of CRC in literatures

The heritability of risk to CRC is estimated to be ~35% [10] but only about 5% of CRC cases can be attributable to highly penetrant mutations in recognized genes. Recent genome-wide association studies (GWASs) have identified a number of common genetic markers significantly associated with CRC [6,11-13]. However, most of these GWAS results have been from populations of European descent. In any GWAS results, the risk associated with any one marker is individually modest, because these markers are rarely causal but merely tag regions haplotypes spanning chromosomal regions. Thus, predicted risks for individuals tend to be very modest and rarely exceed thresholds that would trigger any clinical intervention, and at best these predicted risk might be useful for identifying sub-groups of high-risk subjects carrying multiple risk alleles. Companies such as DeCODEme and 23andme include panels of common SNPs in their testing panels and report predicted risk for complex diseases such as CRC, yet research suggests any prediction based on genetic markers identified through genome-wide studies is of questionable clinical utility [6].

### Present study findings

During the follow-up period which included 633,210 person-year coverage, 258 incident CRC cases (196 men and 62 women) occurred. This case-cohort study evaluated the ability to predict risk based on TRS alone, and these plus a GRS which aggregates information from 7 genetic markers shown to be associated with risk of CRC in Koreans. While most genetic epidemiologic studies have focused on the combined outcome CRC (colon or rectal cancer), but showed less improvement for CRC and colon cancer alone in our Korean sub-cohort study. The rectal cancer prediction model using both TRS and GRS had an increased AUROC by about 3% compared to the AUC from a TRS model (Table 4). The prediction model for rectal cancer alone showed a substantial increase in NRI of about 41%.

We set out to develop and validate CRC risk prediction models and assess their performance in profiling individual genetic risk of CRC in Koreans. We developed models incorporating age, gender, fasting serum glucose, smoking, exercise, family history (FH) and genotype data from 23 common genetic markers reported to significantly associate with CRC in over 47 previous publications. Several of these 23 SNPs (rs3802842, rs4939827, rs6983267, rs10795668, rs961263, rs4779584, and so on) have been well replicated in the scientific literature (Table 3). In Koreans, 7 SNPs (rs3802842, rs4939827, rs6983267, rs10505477, rs10795668, rs961263, rs9929218) among the 23 SNPs were associated with CRC in our sub-cohort based on 258 incident cases. However, some of these 7 SNPs showed positive association with wide 95% confidence intervals.

### CRC versus colon and rectal cancer

Previous GWAS using CRC as the outcome (combining colon and rectal cancer together) reported genome-wide significant associations between risk and multiple SNPs [11-13]. But few studies have considered colon and rectal cancer separately. Some studies of environmental factor argue differences between CRC sub-types may be important [8-9].

When we separated our CRC cases into colon and rectal cancer groups, 7 out of 23 reported risk SNPs showed statistically significant association with CRC and rectal cancer, but not with colon cancer (Table 3). These SNPs showed consistent direction of association and effect size, and the lack of statistical significance could just reflect a loss of power due to smaller sample sizes.

This suggests future studies should also separate colon and rectal cancer rather than just testing only the combined outcome CRC. Also, it raises the question of whether separate prediction models for colon and rectal cancer should be developed.

### TRS versus GRS

In this study of CRC alone, TRS alone showed a strong predictive power of 0.73, and the addition of a GRS failed to show significant contribution or change. In the combined risk models, however, that including both the TRS and GRS, rectal cancer showed the greatest improvement (ROC area change = 3%; NRI = 0.41).

Recently, Dunlop et al. (2013) [6] conducted a ROC analysis of models including genotype data alone or in combination age, gender and FH showed very modest discrimination across the full risk spectrum of risk, with AUC = 0.59 and 0.57 (internal validation) or 0.56 and 0.57 (external validation sets). Their overall positive predictive value fell between 0.51 and 0.71.

The modest performance in individualized CRC risk profiling is consistent with risk prediction studies for other complex diseases (coronary heart disease [14], stroke [15,16], and age-related macular degeneration [17]).

The best predictive performances have been obtained by combining genetic, demographic and environmental variables [17]. In our study, GRS itself showed similar ROC value (~0.6). However, when we combined GRS with traditional risk factors (like age, sex, high fasting glucose, smoking, exercise, and family history) the ROC increased up to 0.74 for predicting CRC, and similar models for rectal cancer showed greater increase.

### Limitation and strength

Major limitations included reliance on self-reported exposures at a single point in time, thus precluding the definitive exclusion of potential misclassification. The statistical power of the current study is modest, as genotyping was performed on a limited sample size of CRC cases and controls. A strong point of our study is the case-cohort design drawn from an underlying large prospective cohort. Case identifications were performed by record linkage to the national cancer registry with verification.

## Conclusion

In conclusion, findings in this current study provide some evidence of improved prediction for CRC in models combining traditional and genetic risk factors. This emphasizes both genetic and traditional factors associated with CRC should be considered when predicting risk.

## Methods

### Study subjects

We have used data on the Korean Metabolic Syndrome Research Initiative in Seoul, initiated in 2005. We have labeled this study as the Korean Cancer Prevention Study-II (KCPS-II). A full description of KCPS-II has been previously published [9,18]. Study members were recruited from participants in routine health assessments at health promotion centers in Seoul and GyeongGi province, South Korea, between 2004 and 2011. Twenty one centers holding electronic health records agreed to linkage of participants’ records to national cancer registry for monitoring of cancer events. The initial study population included 190,332 individuals (112,852 men, 77,480 women), aged 20-94 years. About 90% of participants were enrolled between 2005 and 2008, and the remaining were enrolled prior to or after this period. We have acquired both written consent forms and blood samples from 157,526 participants. Among the total 157,526 participants, 174 participants who reported of having prevalent CRC were excluded. In addition, 11,510 participants who had missing values on body mass index, fasting blood glucose, total cholesterol, systolic blood pressure, smoking status, alcohol drinking, and exercise were excluded. Follow up of participants through December 2011, identified 258 out of these 145,842 participants as incident cases of colorectal cancer.

For the case-cohort study, we selected a sub-cohort as a 1% random sample of all participants. Two of 1,514 randomly selected participants were found to be diagnosed with CRC from our sub-cohort study, while 173 CRC cases were verified outside the sub-cohort. In short, a total of 1,685 additional participants (1,514 plus 173 participants minus 2 participants) were included in our case-cohort study design. Until 2012, the actual number of CRC patients eligible for genetic testing was 173 among all known CRC cases 258. The remaining 85 CRC patients will be tested during the next phase of our study. The Institutional Review Board of Yonsei University reviewed and approved this study.

### Traditional risk score

To develop the traditional risk score (TRS), Cox proportional hazards regression models were fitted first to a basic set of classical risk factors: age, sex, smoking status, fasting serum glucose, family history of colorectal cancer. The TRS algorithm is given in online Additional file 1.

### SNP genotyping

Twenty-three single-nucleotide polymorphisms (rs3802842, rs4444235, rs4939827, rs6983267GG, rs10505477, rs10795668, rs11169552, rs6687758, rs7014346, rs11903757, rs3217810, rs10411210, rs961253, rs6691170, rs9929218, rs10911251, rs7758229, rs59336, rs3217901, rs10936599, rs647161, rs7136702TT, rs4779584) identified in previous 47 studies were genotyped (Table 3 and Additional file 2: Table S1). DNA was isolated from peripheral blood of participants and genotyped at DNA Link Inc. (Seoul, Korea). The genotyping was performed using SNP type assay (Fluidigm, San Francisco, CA, USA) following the manufacturer’s recommendation. Genomic DNA flanking these SNPs of interest was amplified with PCR reaction with STA primer set and Qiagen 2X Mutiplex PCR Master Mix (Qiagen) in 5 microliter reaction volume, containing 60 ng of genomic DNA. PCR reactions were carried out as follows: 15 min at 95°C for 1 cycle, and 14 cycles on 95°C for 15 s and 60°C for 4 min. After amplification, the the STA products were diluted 1:100 in DNA Suspension Buffer. A 2.5 microliter of the diluted STA products were added to a Sample Pre-Mix containing 3 microliter of 2X Fast Probe Master Mix, 0.3 microliter of the SNP type 20X Sample Loading Reagent, 0.1 microliter of the SNP type Reagent, and 0.036 microliter of the ROX. After the Assay Pre-Mix and the Sample Pre-Mix were loaded into the 48.48 Dynamic Array, SNP type assay reaction was carried out. Analysis was carried out using Fluidigm SNP Genotyping Analysis software (version 4.0.1; Fluidigm). Internal quality control (QC) measures were employed to ensure accuracy of the data. A total of 1,685 individuals were genotyped on this platform.

### Anthropometric measurements

Each participant was interviewed using a structured questionnaire to collect information on smoking status and alcohol consumption as well as demographic characteristics, such as age, gender, and family history of various diseases. Cigarette smoking was classified into never smokers, ex-smokers, and current smokers. Alcohol consumption was divided into nondrinkers and current drinkers. Regular physical activity was tracked as either “yes” or “no”. Participant height and weight were measured while the participants were wearing light clothing. Body mass index (BMI) was calculated by dividing the weight (kg) by the square height (m^2^). Systolic and diastolic blood pressures were measured after a rest period of at least 15 min.

### SNP selection and GRS calculation

Each SNP in this study was assumed to be associated with risk following an additive genetic model, which is considered to be generally robust even when the true genetic model is not known or may be incorrectly specified [19]. The GRS was created by two methods: a simple count method (count GRS) and a weighted method (weighted GRS) [14,20]. Both methods assumed each SNP to be independently associated with the risk of CRC (i.e. no interaction). We assumed an additive genetic model for each SNP, applying a linear weighting of 0, 1, or 2 to genotypes containing 0, 1, or 2 of the reported risk alleles, respectively. This count model assumes each SNP in the panel contributes equally to the risk for CRC and was calculated by summing the values for each SNP. The weighted GRS was calculated by multiplying each estimated beta-coefficient by the number of corresponding risk alleles (0, 1, or 2).

In this study, traditional risk factor score (TRS) combined information on 6 risk factors: age, sex, fasting serum glucose, smoking status, exercise status, and family history of CRC.

### Outcome classification

The principle outcome variable was incidence of CRC (n = 258 in whole participants, n = 173 in the sub-cohort), based on data from the national cancer registry. According to the International Classification of Diseases, Tenth Revision (ICD-10), CRC was coded as C18-C20 (C18 for colon, C19 for rectosigmoid, and C20 rectum) [21].

### Statistical analysis

All statistical tests were two-sided, and statistical significance was determined as p<0.05. To evaluate general characteristics of the study population, means and standard deviations (SD) were calculated, and frequencies of cigarette smoking, alcohol consumption, and physical activity was determined. A *χ*^2^ goodness-of-fit test was used to assess whether SNPs were in Hardy-Weinberg Equilibrium and to determine differences in genotype frequencies between CRC cases and controls. The GRS was categorized into quartiles. CRC risk associated with any one genotype was estimated as OR and 95% confidence interval (CI), and was computed using logistic regression under an additive genetic model. We also used receiver operating characteristic (ROC) curve analysis and calculated the area under the curve (AUC; also known as the C statistic) and the continuous net reclassification index (NRI) to evaluate the discrimination power of a CRC risk model. Finally, Cox proportional hazards models were used to estimate the effect of GRS and TRS on CRC risk in our case-cohort design.

### Availability of supporting data

The data set supporting the results of this article is available in the LabArchives, in https://mynotebook.labarchives.com/.

## Additional files

Additional file 1:
**The traditional risk score (TRS).**
Additional file 2: Table S1. Colorectal cancer related 47 references selected for the present study.

## Abbreviations

GRS: Genetic risk score
TRS: Traditional risk score
CRC: Colorectal cancer
OR: Odd ratio
CI: Confidence interval
SNP: Single nucleotide polymorphisms
