# Supplementary material for: A colorectal cancer prediction model using traditional and genetic risk scores in Koreans
Source: BMC Genet. 2015 May 9;16:49. doi: 10.1186/s12863-015-0207-y (PMC4425895; doi:10.1186/s12863-015-0207-y)
Supplement: Additional file 1: — The traditional risk score (TRS). [file 12863_2015_207_MOESM1_ESM.docx]

Additional file 1 The traditional risk score (TRS)

In Table 2, hazard ratios (exponentiated regression coefficients) are given. From these, a linear function was constructed to produce the risk score.

For whole cohort participants, the TRS is defined using the following steps:

x=0.068*(AGE-41.08)-0.436*(SEX-1.38)+0.374*(EXSMOK-0.18)+0.248*(CUSMOK-0.29)-0.099*(EXER1-0.59)+0.594*(FSG10-4.499)+0.876*(FCOL-0.023).

y=exp(x). TRS=(1-0.9965**y).

For sub cohort participants, the TRS is defined using the following steps:

x=0.080*(AGE-41.07)-0.366*(SEX-1.36)+0.571*(EXSMOK-0.17)+0.377*(CUSMOK-0.30)-0.420*(EXER1-0.62)+0.687*(FSG10-4.494)+1.24*(FCOL-0.023).

y=exp(x); TRS=(1-0.9965**y).

Where EXSMOK is ex smoker, CUSMOK is current smoker, EXER1 is exercise, FSG10 is log transformed fasting serum glucose, and FCOL is family history of colorectal cancer. Finally, the absolute TRS is (1-0.9965**y), where 0.9965 is the baseline survival rate.
