# Supplementary material for: A colorectal cancer prediction model using traditional and genetic risk scores in Koreans
Source: BMC Genet. 2015 May 9;16:49. doi: 10.1186/s12863-015-0207-y (PMC4425895; doi:10.1186/s12863-015-0207-y)
Supplement: Additional file 2: Table S1. — Colorectal cancer related 47 references selected for the present study. [file 12863_2015_207_MOESM2_ESM.docx]

Additional file 2: Table S1 Colorectal cancer related 47 references selected for the present study

| No | First Author | Title | Journal | CHR | SNPs |
| --- | --- | --- | --- | --- | --- |
| 1 | Houlston RS | Meta-analysis of three genome-wide association studies identifies susceptibility loci for colorectal cancer at 1q41, 3q26.2, 12q13.13 and 20q13.33 | Nat Genet. 2010;42(11):973-7 | 1 | rs6687758, rs6691170 |
|  |  |  |  | 3 | rs10936599 |
|  |  |  |  | 12 | rs7136702, rs11169552 |
| 2 | Spain SL | Refinement of the associations between risk of colorectal cancer and polymorphisms on chromosomes 1q41 and 12q13.13 | Hum Mol Genet. 2012;21(4):934-46 | 1 | rs6687758, rs6691170 |
| 3 | Peters U | Identification of Genetic Susceptibility Loci for Colorectal Tumors in a Genome-Wide Meta-analysis | Gastroenterology. 2013;144(4):799-807 | 1 | rs10911251 |
|  |  |  |  | 2 | rs11903757 |
|  |  |  |  | 12 | rs3217810, rs59336 |
| 4 | Lubbe SJ | Relationship between 16 susceptibility loci and colorectal cancer phenotype in 3146 patients | Carcinogenesis. 2012;33(1):108-12 | 1 | rs6691170 |
|  |  |  |  | 3 | rs10936599 |
|  |  |  |  | 11 | rs3802842 |
|  |  |  |  | 14 | rs4444235 |
|  |  |  |  | 15 | rs4779584 |
|  |  |  |  | 20 | rs961253 |
| 5 | Figueroa JD | Genome-wide association study identifies multiple loci associated with bladder cancer risk | Hum Mol Genet. 2013 [Epub ahead of print] | 3 | rs10936599 |
| 6 | Chubb D | Common variation at 3q26.2, 6p21.33, 17p11.2 and 22q13.1 influences multiple myeloma risk | Nat Genet. 2013;45(10):1221-5 | 3 | rs10936599 |
| 7 | [Carvajal-Carmona LG](http://www.ncbi.nlm.nih.gov/pubmed/?term=Carvajal-Carmona%20LG%5BAuthor%5D&cauthor=true&cauthor_uid=22999960) | Much of the genetic risk of colorectal cancer is likely to be mediated through susceptibility to adenomas | Gastroenterology. 2013;144(1):53-5 | 3 | rs10936599 |
|  |  |  |  | 8 | rs6983267 |
|  |  |  |  | 10 | rs10795668 |
|  |  |  |  | 11 | rs3802842 |
|  |  |  |  | 14 | rs4444235 |
|  |  |  |  | 18 | rs4939827 |
|  |  |  |  | 20 | rs961253 |
| 8 | Jones AM | TERC polymorphisms are associated both with susceptibility to colorectal cancer and with longer telomeres | Gut. 2012 Feb;61(2):248-54 | 3 | rs10936599 |
| 9 | Jia WH | Genome-wide association analyses in East Asians identify new susceptibility loci for colorectal cancer | Nat Genet. 2013;45(2):191-6 | 5 | rs647161 |
| 10 | [Cui R](http://www.ncbi.nlm.nih.gov/pubmed/?term=Cui%20R%5BAuthor%5D&cauthor=true&cauthor_uid=21242260) | Common variant in 6q26-q27 is associated with distal colon cancer in an Asian population | Gut. 2011;60(6):799-805 | 6 | rs7758229 |
|  |  |  |  | 18 | rs4939827 |
| 11 | [Dai J](http://www.ncbi.nlm.nih.gov/pubmed/?term=Dai%20J%5BAuthor%5D&cauthor=true&cauthor_uid=22505654) | GWAS-identified colorectal cancer susceptibility loci associated with clinical outcomes | Carcinogenesis. 2012;33(7):1327-31 | 8 | rs10505477, rs6983267 |
|  |  |  |  | 20 | rs961253 |
| 12 | Haerian MS | Association of 8q24.21 loci with the risk of colorectal cancer: a systematic review and meta-analysis | J Gastroenterol Hepatol. 2011;26(10):1475-84 | 8 | rs10505477 |
| 13 | Schafmayer C | Investigation of the colorectal cancer susceptibility region on chromosome 8q24.21 in a large German case-control sample | Int J Cancer. 2009;124(1):75-80 | 8 | rs10505477, rs7014346 |
| 14 | Poynter JN | Variants on 9p24 and 8q24 are associated with risk of colorectal cancer: results from the Colon Cancer Family Registry | Cancer Res. 2007;67(23):11128-32 | 8 | rs10505477 |
| 15 | Ghoussaini M | Multiple loci with different cancer specificities within the 8q24 gene desert | J Natl Cancer Inst. 2008;100(13):962-6 | 8 | rs10505477 |
| 16 | Wang YP | Common variation rs6983267 at 8q24.1 and risk of colorectal adenoma and cancer: evidence based on 31 studies | Tumour Biol. 2013 [Epub ahead of print] | 8 | rs6983267 |
| 17 | Hutter CM | Characterization of gene-environment interactions for colorectal cancer susceptibility loci | Cancer Res. 2012;72(8):2036-44 | 8 | rs6983267 |
| 18 | Hutter CM | Characterization of the association between 8q24 and colon cancer: gene-environment exploration and meta-analysis | BMC Cancer. 2010 Dec 4;10:670 | 8 | rs6983267 |
| 19 | He J | Generalizability and epidemiologic characterization of eleven colorectal cancer GWAS hits in multiple populations | Cancer Epidemiol Biomarkers Prev. 2011;20(1):70-81 | 8 | rs6983267 |
|  |  |  |  | 11 | rs3802842 |
|  |  |  |  | 15 | rs4779584 |
|  |  |  |  | 20 | rs961253 |
| 20 | von Holst S | Association studies on 11 published colorectal cancer risk loci | Br J Cancer. 2010;103(4):575-80 | 8 | rs6983267 |
|  |  |  |  | 10 | rs10795668 |
|  |  |  |  | 15 | rs4779584 |
|  |  |  |  | 16 | rs9929218 |
|  |  |  |  | 19 | rs10411210 |
|  |  |  |  | 20 | rs961253 |
| 21 | Abulí A | Susceptibility genetic variants associated with colorectal cancer risk correlate with cancer phenotype | Gastroenterology. 2010;139(3):788-96 | 8 | rs6983267 |
|  |  |  |  | 16 | rs9929218 |
| 22 | Matsuo K | Association between an 8q24 locus and the risk of colorectal cancer in Japanese | BMC Cancer. 2009;9:379 | 8 | rs6983267 |
| 23 | Schafmayer C | Investigation of the colorectal cancer susceptibility region on chromosome 8q24.21 in a large German case-control sample | Int J Cancer. 2009;124(1):75-80 | 8 | rs6983267 |
| 24 | Yeager M | Comprehensive resequence analysis of a 136 kb region of human chromosome 8q24 associated with prostate and colon cancers | Hum Genet. 2008;124(2):161-70 | 8 | rs6983267 |
| 25 | Berndt SI | Pooled analysis of genetic variation at chromosome 8q24 and colorectal neoplasia risk | Hum Mol Genet. 2008;17(17):2665-72 | 8 | rs6983267 |
| 26 | Li L | A common 8q24 variant and the risk of colon cancer: a population-based case-control study | Cancer Epidemiol Biomarkers Prev. 2008;17(2):339-42 | 8 | rs6983267 |
| 27 | Poynter JN | Variants on 9p24 and 8q24 are associated with risk of colorectal cancer: results from the Colon Cancer Family Registry | Cancer Res. 2007;67(23):11128-32 | 8 | rs6983267 |
| 28 | Tomlinson I | A genome-wide association scan of tag SNPs identifies a susceptibility variant for colorectal cancer at 8q24.21 | Nat Genet. 2007;39(8):984-8 | 8 | rs6983267 |
| 29 | Tenesa A | Genome-wide association scan identifies a colorectal cancer susceptibility locus on 11q23 and replicates risk loci at 8q24 and 18q21 | Nat Genet. 2008;40(5):631-7 | 8 | rs7014346 |
|  |  |  |  | 11 | rs3802842 |
|  |  |  |  | 18 | rs4939827 |
| 30 | Hong SN | Colorectal Cancer-Susceptibility Single Nucleotide Polymorphisms in Korean Population | J Gastroenterol Hepatol. 2013 [Epub ahead of print] | 10 | rs10795668 |
|  |  |  |  | 15 | rs4779584 |
|  |  |  |  | 18 | rs4939827 |
| 31 | Abulí A | Genetic susceptibility variants associated with colorectal cancer prognosis | Carcinogenesis. 2013;34(10):2286-91 | 10 | rs10795668 |
|  |  |  |  | 16 | rs9929218 |
| 32 | Giráldez MD | Susceptibility genetic variants associated with early-onset colorectal cancer | Carcinogenesis. 2012;33(3):613-9 | 10 | rs10795668 |
|  |  |  |  | 11 | rs3802842 |
|  |  |  |  | 14 | rs4444235 |
|  |  |  |  | 15 | rs4779584 |
|  |  |  |  | 20 | rs961253 |
| 33 | Ho JW | Replication study of SNP associations for colorectal cancer in Hong Kong Chinese | Br J Cancer. 2011;104(2):369-75 | 10 | rs10795668 |
|  |  |  |  | 15 | rs4779584 |
| 34 | Xiong F | Risk of genome-wide association study-identified genetic variants for colorectal cancer in a Chinese population | Cancer Epidemiol Biomarkers Prev. 2010;19(7):1855-61 | 10 | rs10795668 |
|  |  |  |  | 11 | rs3802842 |
|  |  |  |  | 18 | rs4939827 |
|  |  |  |  | 20 | rs961253 |
| 35 | Tomlinson IP | A genome-wide association study identifies colorectal cancer susceptibility loci on chromosomes 10p14 and 8q23.3 | Nat Genet. 2008;40(5):623-30 | 10 | rs10795668 |
| 36 | Hes FJ | Colorectal cancer risk variants on 11q23 and 15q13 are associated with unexplained adenomatous polyposis | J Med Genet. 2014;51(1):55-60 | 11 | rs3802842 |
|  |  |  |  | 15 | rs4779584 |
| 37 | Song H | Effects of common germ-line genetic variation in cell cycle genes on ovarian cancer survival | Clin Cancer Res. 2008;14(4):1090-5 | 12 | rs3217901 |
| 38 | COGEN study | Meta-analysis of genome-wide association data identifies four new susceptibility loci for colorectal cancer | Nat Genet. 2008;40(12):1426-35 | 14 | rs4444235 |
|  |  |  |  | 16 | rs9929218 |
|  |  |  |  | 19 | rs10411210 |
|  |  |  |  | 20 | rs961253 |
| 39 | Xing J | GWAS-identified colorectal cancer susceptibility locus associates with disease prognosis | Eur J Cancer. 2011;47(11):1699-707 | 15 | rs4779584 |
| 40 | Siegert S | Genome-wide investigation of gene-environment interactions in colorectal cancer | Hum Genet. 2013;132(2):219-31 | 16 | rs9929218 |
| 41 | Zhang B | Genome-wide association study identifies a new SMAD7 risk variant associated with colorectal cancer risk in East Asians | Int J Cancer. 2014 [Epub ahead of print] | 18 | rs4939827 |
| 42 | Garcia-Albeniz X | Phenotypic and tumor molecular characterization of colorectal cancer in relation to a susceptibility SMAD7 variant associated with survival | Carcinogenesis. 2013;34(2):292-8 | 18 | rs4939827 |
| 43 | Phipps AI | Association between colorectal cancer susceptibility loci and survival time after diagnosis with colorectal cancer | Gastroenterology. 2012;143(1):51-4 | 18 | rs4939827 |
| 44 | Slattery ML | Increased risk of colon cancer associated with a genetic polymorphism of SMAD7 | Cancer Res. 2010;70(4):1479-85 | 18 | rs4939827 |
| 45 | Thompson CL | Association of common genetic variants in SMAD7 and risk of colon cancer | Carcinogenesis. 2009;30(6):982-6 | 18 | rs4939827 |
| 46 | Curtin K | Meta association of colorectal cancer confirms risk alleles at 8q24 and 18q21 | Cancer Epidemiol Biomarkers Prev. 2009;18(2):616-21 | 18 | rs4939827 |
| 47 | Fernandez-Rozadilla C | BMP2/BMP4 colorectal cancer susceptibility loci in northern and southern European populations | Carcinogenesis. 2013;34(2):314-8 | 20 | rs961253 |

CHR: chromosome, SNPs: single nucleotide polymorphisms
